# Genomic Organization of Zebrafish microRNAs

**DOI:** 10.1186/1471-2164-9-253

**Published:** 2008-05-29

**Authors:** Elizabeth J Thatcher, Jordan Bond, Ima Paydar, James G Patton

**Affiliations:** 1 Department of Biological Sciences, Vanderbilt University, Nashville, TN, USA

## Abstract

**Background:**

microRNAs (miRNAs) are small (~22 nt) non-coding RNAs that regulate cell movement, specification, and development. Expression of miRNAs is highly regulated, both spatially and temporally. Based on direct cloning, sequence conservation, and predicted secondary structures, a large number of miRNAs have been identified in higher eukaryotic genomes but whether these RNAs are simply a subset of a much larger number of noncoding RNA families is unknown. This is especially true in zebrafish where genome sequencing and annotation is not yet complete.

**Results:**

We analyzed the zebrafish genome to identify the number and location of proven and predicted miRNAs resulting in the identification of 35 new miRNAs. We then grouped all 415 zebrafish miRNAs into families based on seed sequence identity as a means to identify possible functional redundancy. Based on genomic location and expression analysis, we also identified those miRNAs that are likely to be encoded as part of polycistronic transcripts. Lastly, as a resource, we compiled existing zebrafish miRNA expression data and, where possible, listed all experimentally proven mRNA targets.

**Conclusion:**

Current analysis indicates the zebrafish genome encodes 415 miRNAs which can be grouped into 44 families. The largest of these families (the miR-430 family) contains 72 members largely clustered in two main locations along chromosome 4. Thus far, most zebrafish miRNAs exhibit tissue specific patterns of expression.

## Background

As the transcriptional landscapes of eukaryotic genomes are defined, it appears that overall transcription is much more prevalent than previously thought, perhaps by as much as 10-fold greater than that needed to generate mRNAs encoding the majority of protein coding genes [1]. Abundant noncoding RNAs, both short and long, have been identified but for the most part their functional significance remains unknown. Among recently discovered small RNAs, the best characterized thus far are microRNAs (miRNAs) [2,3]. Direct cloning strategies and bioinformatic predictions based on the presence of conserved hairpin structures and sequences have suggested that animal genomes encode hundreds, perhaps thousands, of miRNAs [4-7]. Cell movement, specification, and development are regulated, in part, by miRNAs, consistent with the fact that expression of these RNAs is highly regulated in a tissue and time-specific manner. miRNAs originate from RNA Polymerase II transcripts [8] requiring processing by the RNase III-like enzyme, Drosha before nuclear export. From the large primary transcripts, Drosha releases hairpins that are ~70 nucleotides long with extensive pairing of approximately 28 base pairs in the stem [9]. Hairpin precursors are exported from the nucleus in a RAN-GTP dependent manner using Exportin 5 [10,11]. In the cytoplasm, miRNA precursors are further processed by a second RNase III-like enzyme, Dicer, releasing mature miRNA duplexes of ~22 nucleotides [12-14]. Typically, only one strand of the duplex pairs with a target mRNA as part of a larger dynamic ribonucleoprotein complex referred to as the RNA Induced Silencing Complex (RISC). Argonuate proteins are key components of RISCs and are thought to play an important role in whether the target mRNA is subject to translational repression or cleavage followed by degradation [15].

miRNAs usually pair with sequence elements (miRNA Recognition Elements; MREs) within the 3' UTR of their target mRNAs but there have been limited examples of pairing in the 5' UTR [16]. Since miRNAs usually pair with incomplete complementarity to their targets, bioinformatic approaches to identify targets are limited and functional analysis is required to prove mRNA:miRNA interactions. Because of this challenge, only a small number of targets have been experimentally proven. Further, since each miRNA can target multiple mRNAs and a single mRNA can be targeted by multiple miRNAs, significant work remains to characterize the full range of miRNA function [17,18].

Zebrafish have proven to be a valuable model system to investigate miRNA function and characterize miRNA:mRNA interactions. Since the creation of active miRNAs requires cleavage by Dicer, zygotic Dicer mutants and maternal zygotic Dicer mutants have helped define the role of miRNAs during development [19,20]. Zygotic Dicer null mutants live approximately 14 days, because there is sufficient maternal Dicer mRNA deposited into the oocyte [19]. Maternal zygotic Dicer mutants exhibit more severe developmental defects and die after only 7 days [20]. Thus, the regulation of miRNA expression is critical for early zebrafish development. For example, *miR-214 *is required for proper muscle formation, miR-375 is needed for pancreatic islet development, and the large *miR-430 *family is needed for deadenylation and clearance of maternal mRNAs at the midblastula transition [21-23].

External fertilization, fast development, and ease of genetic manipulation make zebrafish a powerful system to study vertebrate development and analyze miRNAs. To facilitate this work, microarrays and *in situ *hybridization experiments have provided a wealth of knowledge regarding temporal and spatial expression of miRNAs during zebrafish development [13,14,24,25]. Small RNA cloning coupled with bioinformatic prediction have enabled the identification of many zebrafish miRNAs but since genome sequencing and annotation is not yet complete, the exact number of miRNAs remains to be determined. Here, we have utilized existing databases and newly available genomic sequence information to identify and catalog all known and predicted zebrafish miRNAs.

## Results

### Zebrafish miRNAs and Genomic Locations

Currently, the microRNA registry lists 380 miRNAs in the zebrafish genome [26]. Inclusion in the Registry implies experimental validation through direct sequencing, northern blots, or microarray approaches. As the zebrafish genome is not fully sequenced and annotated, it is likely that the sum total of zebrafish miRNAs will be larger than currently contained within the miRNA Registry. We took the approach to use sequence conservation across species and prediction algorithms to identify additional zebrafish miRNAs. Such algorithms have been applied to the human, *C. elegans*, and pufferfish genomes to identify miRNAs by searching for conserved hairpin structures of 60–100 nucleotides containing no branches with only a few mismatches or bulges [7]. Using MiRScan, the Sanger microRNA registry, and RNAfold, we analyzed the zebrafish genome and identified 35 new zebrafish miRNAs (Table 1). First, mature miRNA sequences from mouse and human were compared to the zebrafish genome using BLAST to identify conserved sequences. The criteria for inclusion required perfect seed sequences and less than 3–4 mismatches in the 3' ends. Next, candidate zebrafish miRNAs were examined for their genomic position to determine whether they resided in conserved genes or transcripts compared to mouse and human. Sequences adjacent to candidate miRNAs were then subjected to RNAfold to identify the presence of conserved hairpin structures. Hairpin structures and sequences were then compared across human, mouse, and zebrafish using MiRscan and Clustal W to confirm the presence of conserved sequences resembling miRNAs. Such analyses resulted in the identification of 35 new potential miRNAs bringing the total to 415 when combined with previously documented miRNAs and including those cases where both strands are utilized. We also compared the new predicted miRNAs to the Takfugu rubripes (fugu) genome and found that 7 of the new miRNAs were conserved between the two genomes. Since the fugu genome is considerably smaller than the zebrafish genome and may lack a number of elements including noncoding RNAs, we focused more extensively on sequence conservation across the zebrafish, human, and mouse genomes.

**Table 1:** Newly Identified Zebrafish miRNAs.

**Name**	**Chromosome**	**Conserved**	**Name**	**Chromosome**	**Conserved**
miR-466a-3p	2	mmu	miR-467a*-9	8	mmu
miR-466b-5p	24	mmu	miR-467d*	11	mmu
miR-466c-1	12	mmu	miR-467e*-1	16	mmu
miR-466c-2	12	mmu	miR-467e*-2	10	mmu
miR-466d	17	mmu, fru	miR-469a	16	mmu
miR-466h-1	1	mmu, fru	miR-705	9	mmu
miR-466h-2	1	mmu, fru	miR-710	17	mmu
miR-466h-3	1	mmu, fru	miR-718	14	mmu
miR-466h-4	1	mmu, fru	miR-741	7	mmu
miR-466h-5	24	mmu, fru	miR-743b-5p	25	mmu
miR-467a*-1	13	mmu	miR-758	18	mmu
miR-467a*-2	4	mmu	miR-759	8	mmu
miR-467a*-3	21	mmu	miR-760	8	mmu, fru
miR-467a*-4	12	mmu	miR-872	3	mmu
miR-467a*-5	18	mmu	miR-876-3p	24	mmu
miR-467a*-6	12	mmu	miR-876-5p	20	mmu
miR-467a*-7	12	mmu	miR-883b-3p	3	mmu
miR-467a*-8	24	mmu			

Conserved mouse miRNA sequences within the microRNA Registry were used to conduct BLAST searches of the existing zebrafish and fugu genomes. Candidate sequences (>80% sequency identity) were further analyzed using mFold and MiRscan. For those sequences that matched miRNAs from other vertebrate species, the same numbering system was applied resulting in the miRNAs listed above. miRNAs with an sterisk indicate that both strands of the dsRNA precursor are packaged into RNA Induced Silencing Complexes (RISCs). (fru: fugu, mmu: mouse)

**Figure 1:**
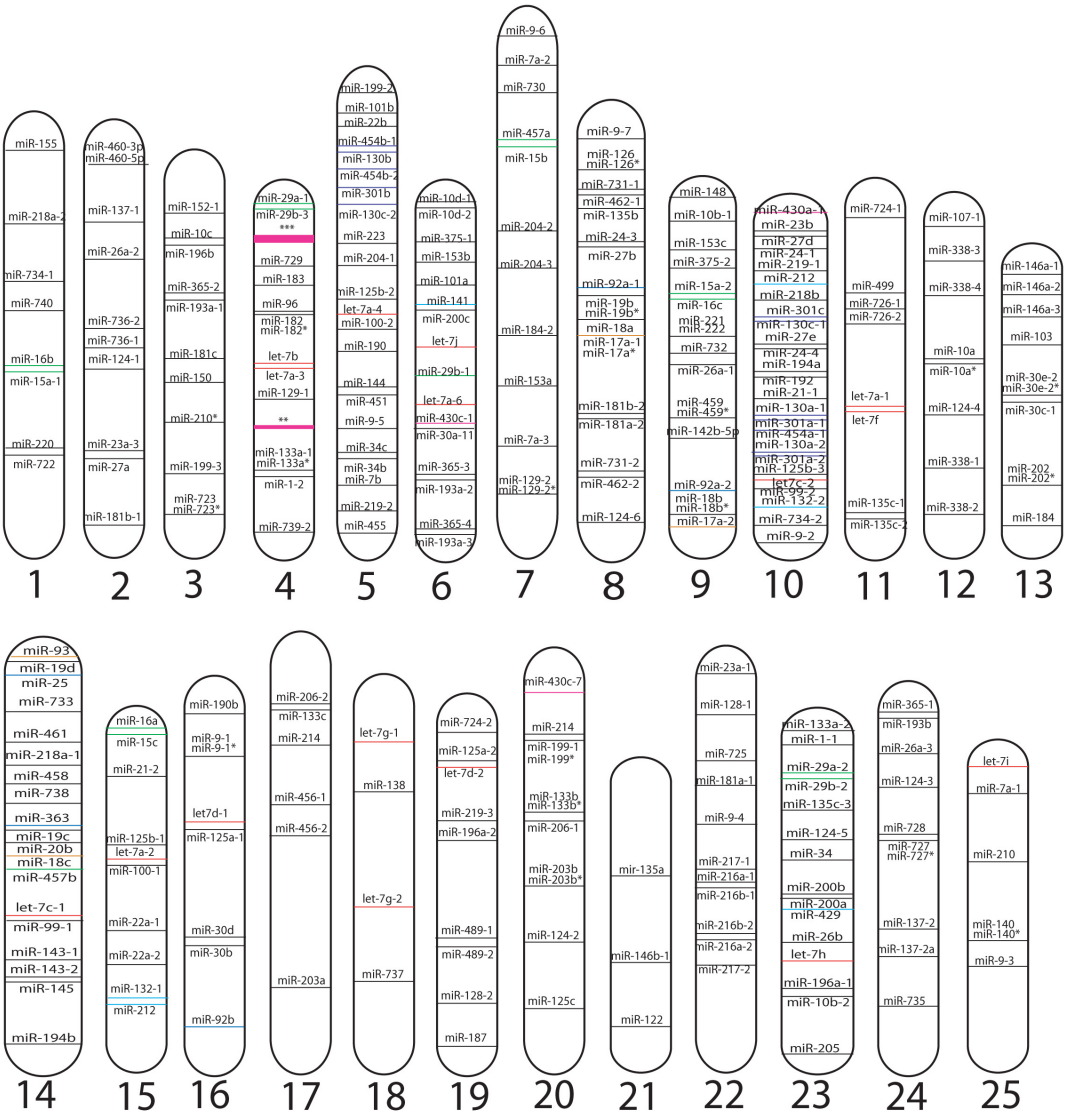
**Chromosomal Location of Zebrafish miRNAs**. The relative locations of individual miRNAs are shown across the 25 zebrafish chromosomes. miRNA families are denoted by different colors.

**Figure 2:**
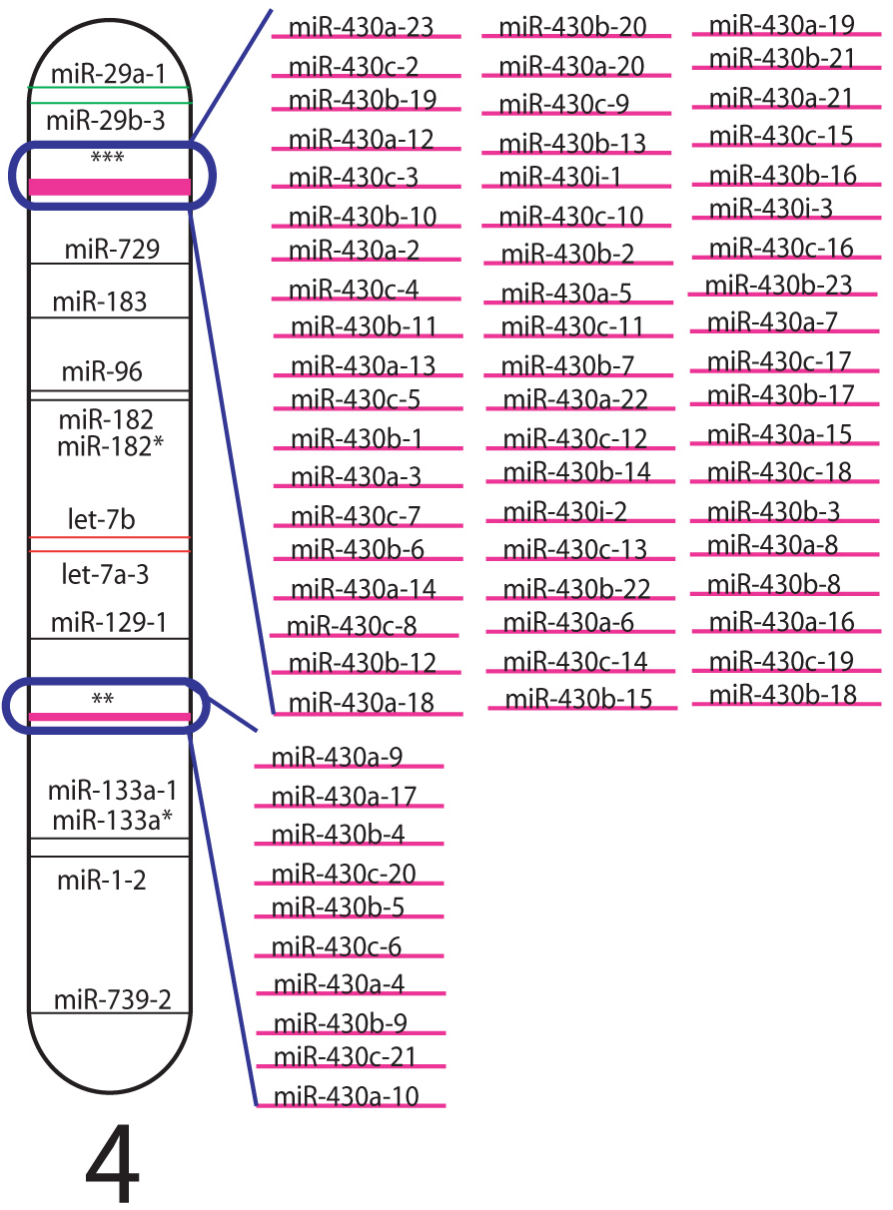
**Clustered Zebrafish miRNAs**. The *miR-430 *family has two large clusters on chromosome 4 consisting of 57 and 10 copies, respectively.

Next, we determined the chromosomal location of all miRNA genes in zebrafish. Figure 1 shows the location of miRNAs across the 25 zebrafish chromosomes. Some chromosomes are relative miRNA deserts (chromosomes 18, 21, and 25) whereas others encode large numbers of miRNAs (chromosomes 4, 5 10, 14). Strikingly, the *miR-430 *family contains two large clusters of 10 and 57 genes on chromosome 4 (Figure 2). Such large copy numbers are consistent with the function of the *miR-430 *family to target and degrade the wide variety and number of maternally deposited mRNAs coincident with zygotic transcription at the midblastula transition [22]. miRNA genes that could not yet be mapped were listed by sequencing scaffold numbers rather than chromosomal assignment and are included in Additional File 1. This table also contains current information for those miRNAs whose temporal and spatial expression patterns have been reported as well as a listing of validated mRNA targets.

Based on chromosomal location and additional genomic analysis, we were able to tabulate those miRNAs that are encoded as distinct transcripts versus those that are encoded within the introns or exons of other genes (Table 2). In mammals, approximately 50% of miRNAs are encoded within the introns of protein coding genes whereas the remainder are independently synthesized as either mono- or poly-cistronic noncoding transcripts [27-29]. For zebrafish, most miRNAs (~86%) are found in intergenic regions while only 12% exist within the introns of other genes (Table 2). We defined introns and exons only in the context of protein coding genes which may account for part of the differences between reported mammalian and zebrafish percentages. Some miRNAs are encoded within "host" genes that are split into exons and introns and therefore subject to splicing but we elected to refer to these miRNAs as independent transcripts since no proteins are apparently encoded in the host gene (for example, ENSESTG00000015836; [27]). As more genes are annotated within the zebrafish genome, some of the miRNAs we defined as intergenic may in fact reside within other RNA Polymerase II transcribed genes.

**Table 2:** Characteristics of Zebrafish miRNAs.

	**Number**	**Percentage**
Exonic	2	0.5%
Intronic	53	12.8%
Intergenic	356	85.8%
Monocistronic	219	52.8%
Polycistronic	196	47.2%

The location of zebrafish miRNAs was analyzed to determine whether individual miRNAs are encoded within exons, introns, or as independent intergenic transcripts. Polycistronic transcripts indicates that two or more miRNAs are located within 3 kb of one another.

### Zebrafish miRNA Transcriptional Units

Since miRNAs can originate from either mono- or poly-cistronic transcripts, we next identified zebrafish miRNAs that are closely linked in the genome and attempted to classify miRNAs as part of polycistronic transcripts based on position and expression analysis [24]. We decided to include miRNAs as part of polycistronic transcripts if the mature sequences are within 3 kb and our array data indicated similar or identical expression patterns. The decision to use 3 kb as a distance between miRNAs was arbitrary and is almost certainly an underestimate. Nevertheless, as shown in Table 3, a large number (~50%) of zebrafish miRNAs were found to reside within 3 kb of another miRNA suggesting that zebrafish miRNAs are extensively encoded within polycistronic transcripts. Recent analysis of miRNA target specificity determinants suggested that mRNAs containing multiple MREs that can be targeted by co-expressed miRNAs increases the likelihood of targeting [30]. Thus, knowing which miRNAs are transcribed as part of polycistronic transcripts will help to identify potential targets for a given miRNA.

**Table 3:** Polycistronic Zebrafish miRNAs.

**Chromosome**	**Transcript Members**
1	miR-16b, miR-15a-1
2	miR-23a-3, miR-27a
3	miR-365-2, miR-193a-1
4	miR-29a-1, miR-29b-3
4	Clustered miR-430 family: 57 members
4	miR-183, miR-96, miR-182*, miR-182
4	let-7b, let-7a-3
4	Clustered miR-430 family: 10 members
4	miR-133a*, miR-133a-1, miR-1-2
5	miR-130b, miR-454b-2
5	miR-301b, miR-130c-2
5	let-7a-4, miR-100-2
5	miR-144, miR-451
5	miR-34c, miR-34b
6	miR-141, miR-200c
6	miR-430c-1, miR-430a-11
6	miR-365-3, miR-193a-2
6	miR-365-4, miR-193a-3
7	miR-457a, miR-15b
8	miR-731-1, miR-462-1
8	miR-92a-1, miR-19b*, miR-19b, miR-20a, miR-19a*, miR-19a, miR-17a-1, miR-17a*
8	miR-181b-2, miR-181a-2
8	miR-731-2, miR-462-2
9	miR-15a-2, miR-16c
9	miR-221, miR-222
9	miR-92a-2, miR-17a-2
10	miR-23b, miR-27d
10	miR-310c, miR-130c
10	miR-194a, miR-192
10	miR-130a-1, miR-301a-1, miR-454a-1
10	miR-130a-2, miR-301a-2
10	let-7c-2, miR-99-2
11	let-7a-1, let-7f
14	miR-93, miR-19d, miR-25
14	miR-363, miR-19c, miR-20b, miR-18c
14	let-7c-1, miR-99-1
15	miR-16a, miR-15c
15	let-7a-2, miR-100-1
16	miR-30d, miR-30b
20	miR-214, miR-199*, miR-199-1
20	miR-133b, miR-133b*, miR-206-1
22	miR-217-1, miR-216a-1, miR-216b-1
22	miR-216b-2, miR-216a-2, miR-217-2
23	miR-29a-2, miR-29b-2
23	miR-200b, miR-200a, miR-429
Zv6_NA1101	miR-23a-2, miR-27c, miR-27c*
Zv6_scaffold3754	let-7e, let-7a-5

The identity of individual miRNAs that reside within 3 kb of one another is shown along with chromosomal assignment. Predicted polycistronic transcripts were compared to known expression data to determine if the miRNAs had similar expression profiles.

### miRNA Families

The seed region is defined as nucleotides 2–7 from the 5' end of the miRNA and is a key determinant in pairing with target mRNAs [17]. miRNAs can be grouped based on sequence identity within the seed region with the prediction that specific mRNAs can be targeted by multiple miRNAs provided these miRNAs contain identical seed sequences even if other downstream nucleotides vary. We therefore examined zebrafish miRNAs and placed those with identical seed sequences in the same family (Table 4). As above, the best example of this is the *miR-430 *family which is the most abundant zebrafish miRNA discovered thus far. There are 5 members of the family with over 90 copies apparently transcribed as part of 4 different RNAs. Family members bind to a common sequence found in the 3' UTR of maternal transcripts [22].

**Table 4:** Zebrafish miRNA Families.

**Seed**	**Family Members**
AAAGUG	mir-93, miR-20b, miR-17a, miR-20a
GGAAGA	miR-7a, miR-7b
UAAGAC	miR-499, miR-736
CAGGAA	miR-461, miR-459
AAGUGC	miR-430a, miR-430b, miR-430c, miR-130j, miR-430i
AUUGCA	miR-363, miR25, miR-92b, miR-92a
AGGCCG	miR-34b, miR-15a*, miR-456
GGCAGU	miR-34, miR-34c
GUAAAC	miR-30a, miR-30b, miR-30c, miR-30d, miR-30e
UCACAG	miR-27a, miR-27b, miR-27c, miR-27d, miR-27e
UCAAGU	miR-26a, miR-27b
UCACAU	miR-23a, miR-23b
AGCUGC	miR-22a, miR-22b
GCUACA	miR-222, miR-221
UGUGCU	miR-218a, miR-218b
AAUCUC	miR-216a, miR-216b
UGAAAU	miR-203a, miR-203b
AAUACU	miR-200b, miR-200c, miR-429
GUGCAA	miR-19a, miR-19b, miR-19c, miR-19d
CCAUUG	miR-199, miR-181a*
AGGUAG	miR-196b, miR-196a
GUAACA	miR-194a, miR-194b
ACUGGC	miR-193b, miR-193a
GAUAUG	miR-190, miR-190b
AAGGUG	miR-18a, miR-18c, miR18b
UUGGCA	miR-182, miR-96
ACAUUC	miR-181, miR-181b, miR-181c
UGCAUA	miR-153a, miR-153b, miR-153c
CAGUGC	miR-152, miR-148
GAGAAC	miR-146b, miR-146a
AUAAAG	miR-142a-5p, miR-142b-5p
AACAGU	miR-141, miR-200a, miR-132, miR-212
AUGGGU	miR-135, miR-135b, miR-135a, miR-729
UUGGUC	miR-133a, miR-133b, miR-133c
GCUGGU	miR-133a*, miR-138
AGUGCA	miR-130b, miR-301a, miR-301b, miR-301c, miR-130a, miR-130c, miR-454a, miR-454b
CCCUGA	miR-125c, miR-125b, miR-125a
ACCCUG	miR-10d, miR-10b, miR-10c
GCAGCA	miR-107, miR-103
ACAGUA	miR-101b, miR-101a, miR-199*, miR-144
ACCCGU	miR-100, miR-99
GGAAUG	miR-1, miR-206
GAGGUA	let-7a, let-7b, let-7c, let-7d, let-7e, let-7f, let-7g, let-7h, let-7i, let-7j
AGCAGC	miR-29b, miR-29a, miR-457b, miR-457a, miR-15a, miR-15b, miR-15c, miR-16b, miR-16a, miR-16c

miRNA with identical seed sequences were grouped into distinct families are shown.

## Discussion

As more and more miRNAs are identified, it has become ever more apparent that understanding global gene regulation requires identifying the targets of every miRNA and the functional consequences of such targeting. Microarrays have been used extensively to determine global miRNA expression patterns. Complementing such analyses with *in situ *localization of miRNAs greatly facilitates testing of candidate target genes during zebrafish development [20,24,25,31]. Because of imperfect miRNA:mRNA pairing, computer algorithms to identify specific miRNA targets typically produce lists of several hundred candidate genes. Such lengthy lists can be partially refined by integrating spatial and temporal expression data for both miRNAs and their targets. Further, because many miRNAs share identical seed sequences (Table 4), it is important to identify all miRNAs that may target a given mRNA. Here, we have analyzed the existing zebrafish genome to expand the list of miRNAs to 415. We determined the chromosomal location for each miRNA and grouped them into seed sequence families. In addition, we compiled existing expression data and listed validated mRNA targets. Together, Additional File 1 provides an easy to access database that should prove valuable for those interested in understanding the role that miRNAs play in regulating gene expression.

The ease with which gain-of-function and loss-of-function experiments can be conducted in zebrafish makes it an attractive model system to study miRNA function. For loss-of-function experiments, whether in zebrafish, cultured cells, or other model organisms, it is imperative that all members of a given family be effectively knocked down to generate consistent phenotypes. By examining the data in this paper and in Additional File 1, it is possible to quickly determine functional redundancy between one or more miRNAs. Such knowledge will help to design antisense morpholino oligonucleotides when entire miRNA families need to be knocked down [21,23].

## Conclusion

Based on sequence conservation, we identified 35 new zebrafish miRNAs bringing the total number of miRNAs encoded by the zebrafish genome to 415. Bearing in mind that the zebrafish genome is not completely sequenced and annotated, analysis of the existing data suggests that the majority of miRNAs thus far evaluated are encoded as distinct, tissue specific transcripts with an even split between those contained as part of polycistronic transcripts versus those encoded as monocistronic transcripts.

## Methods

To identify new zebrafish miRNAs, existing miRNA sequences from mouse were retrieved from the miRNA registry [26] and compared with the zebrafish and fugu genome using BLAST and Ensemble's Zebrafish or Fugu Genome databases [32,33]. Predicted alignments that contained one or more mismatches within the seed region of the miRNA were discarded whereas no more than 3–4 mismatches were allowed in 3' regions. Resulting sequences were then evaluated for hairpin secondary structures using Vienna RNA Secondary Structure Prediction Program (RNAfold [34]). For those potential miRNAs exhibiting mature miRNA sequence conservation and predicted hairpin structures, MiRscan [7] and ClustalW2 [35] were utilized to compare hairpin precursor sequences to each other and to 50 previously described and highly conserved *C. elegan *miRNAs. Further, predicted zebrafish hairpins were examined to primarily include those located within the same transcriptional unit as their human or mouse counterpart.

Family members were determined strictly by identical seed sequences (2^nd^–7^th ^nts from the 5' end). Intronic, exonic, and intergenic miRNAs were determined by location among predicted genes within Ensemble. Polycistronic family members were classified as being within 3 kb of another known or predicted miRNA and showing similar or identical expression. Zebrafish miRNA expression data was compiled from previously published sources and is included in Additional File 1[13,14,24,25].

## Authors' contributions

EJT, JB, and IP performed all experiments and analyses and created all figures. JGP designed and directed the project and JGP and EJT wrote the paper. All authors read and approved the final manuscript.

## Supplementary Material

Additional file 1 **Zebrafish miRNAs**. Compilation of current zebrafish miRNA data in an excel spreadsheet. Included for each miRNA is its Registry name, genomic name, mature miRNA sequence, seed sequence, miRNA family members, expression data from two sources, chromosome #, mono- vs poly-cistronic transcripts, intron, exon or intergenic location, polycistronic transcript family members, proven targets and sense vs anti-sense. Click here for file
